# Association of *PEDF* polymorphisms with age-related macular degeneration and polypoidal choroidal vasculopathy: a systematic review and meta-analysis

**DOI:** 10.1038/srep09497

**Published:** 2015-03-30

**Authors:** Li Ma, Shu Min Tang, Shi Song Rong, Haoyu Chen, Alvin L. Young, Govindasamy Kumaramanickavel, Chi Pui Pang, Li Jia Chen

**Affiliations:** 1 Department of Ophthalmology and Visual Sciences, The Chinese University of Hong Kong, Hong Kong, China; 2 Department of Ophthalmology and Visual Sciences, Prince of Wales hospital, Hong Kong, China; 3 The Shantou University/Chinese University of Hong Kong Joint Shantou International Eye Center, Shantou, Guangdong Province, China; 4 Narayana Nethralaya, Health City, Bangalore, India

## Abstract

This study assesses the association of the *pigment epithelium-derived factor (PEDF)* gene with age-related macular degeneration (AMD) and polypoidal choroidal vasculopathy (PCV). Publications in MEDLINE and EMBASE up to 21/08/2014 were searched for case-control association studies of *PEDF* with AMD and/or PCV. Reported studies giving adequate genotype and/or allele information were included. Pooled odds ratios (OR) and 95% confidence intervals (CI) of each polymorphism were estimated. Our literature search yielded 297 records. After excluding duplicates and reports with incomplete information, 8 studies were eligible for meta-analysis, involving 2284 AMD patients versus 3416 controls, and 317 PCV patients versus 371 controls. Four *PEDF* polymorphisms were meta-analyzed: rs1136287, rs12150053, rs12948385 and rs9913583, but none was significantly associated with AMD or PCV. The most-investigated polymorphism, rs1136287, had a pooled-OR of 1.02 (95% CI: 0.94–1.11, P = 0.64) for AMD. In subgroup analysis by ethnicity, no significant association was identified. Polymorphisms present in single report showed no association. Therefore, existing data in literature does not support the role of *PEDF* in the genetic susceptibility of AMD and PCV, although replication in specific populations is warranted. Since the pooled-sample size for PCV was small, there is a need of *PEDF* genotyping in larger samples of PCV.

Age-related macular degeneration (AMD) is a degenerative disease at the central region of the retina - the macula, leading to distorted central vision in the early stage and severe visual loss in the late stage. AMD is a leading cause of irreversible visual disability and blindness among elderly in developed countries^1^. The prevalence of early AMD is approximately 8.01% and late AMD (including geographic atrophy and neovascular AMD [nAMD]) 0.37%^2^. The etiology of AMD is multifactorial, with genetic risk factors contributing to the disease development and progression^3^. Genes in the complement pathway, such as complement factor H (*CFH*)^3^,^4^, angiogenesis pathway, such as vascular endothelial growth factor (*VEGF*)^5^, the high-density lipoprotein metabolic pathway, such as cholesteryl ester transfer protein (*CETP*)^6^, and the HtrA serine peptidase 1 (*HTRA1*) gene^7^, have been associated with AMD.

Polypoidal choroidal vasculopathy (PCV), which is considered as a subtype of AMD, could also cause profound loss of central vision. Its prevalence is higher in Asians than in Caucasians^8^. PCV exhibits commonalities with nAMD in that both are choroidal vasculopathy associated with subretinal hemorrhage, scars and fibrosis^8^. However, PCV is characterized by inner choroidal vascular networks ending in polypoidal lesions, while nAMD is characterized by choroidal neovascularization (CNV)^9^,^10^. Moreover, PCV patients are relatively younger, usually lack drusen, and respond well to combined anti-VEGF and photodynamic therapy, while nAMD patients respond well to anti-VEGF monotherapy^11^,^12^. PCV is also a multifactorial disease with genetic susceptibility. Major genes of AMD, such as *CFH*, *HTRA1* and *CETP*, also showed significant associations with PCV^6^. These aspects lead to a question whether PCV is a subtype of, or a distant phenotype from nAMD.

It has been assumed that abnormal vessels are caused by unbalanced stimulators and inhibitors of angiogenesis^13^. An imbalance between VEGF and pigment epithelial derived factor (PEDF) has been demonstrated in the progress of CNV in AMD^14^,^15^. The vascular endothelial growth factor A (VEGF-A) plays an important role in inducing the growth of choroidal new vessels^16^,^17^,^18^, which triggers proliferation and migration of vascular endothelial cells^19^. Anti-VEGF therapies have been widely used to treat both nAMD and PCV. Also, variants in the *VEGF-A* gene have been implicated in the genetic mechanism of AMD and PCV^20^,^21^. A recent meta-analysis of 9 articles with 2281 AMD cases versus 2820 controls revealed that *VEGF-A* rs1413711 and rs833061 increased the risk of AMD^21^.

In contrast to VEGF, the PEDF, a member of the serine proteinase inhibitor family, potently inhibits angiogenesis and regulates choroidal neovascularization in humans^22^,^23^,^24^,^25^. PEDF has been detected in the aqueous humor, vitreous, retina and choroid^26^,^27^. In the retina, PEDF inhibits the proliferation and migration of retinal endothelial cells and vascular permeability induced by VEGF, promotes the apoptosis of endothelial cells and down-regulates the pro-angiogenic factors^23^,^28^,^29^. PEDF is also a highly effective inhibitor of angiogenesis in cell culture and animal models^25^,^30^,^31^,^32^. Decreased vitreous level of PEDF had been associated with the CNV in AMD^33^. PEDF as a potential therapeutic agent has been investigated in animal models of CNV, and it was found that periocular or intravitreal introduction of PEDF could inhibit CNV^22^,^34^,^35^,^36^. Moreover, patients with nAMD were found to have reduced CNV size after a single intravitreal injection of PEDF-expressing adenoviral vector in a phase I clinical trial^37^. These studies altogether suggest that PEDF is an important factor for CNV, and hypothetically the *PEDF* gene is an excellent candidate gene for nAMD.

In 2005, Yamagishi et al. hypothesized that a single nucleotide polymorphism (SNP) in *PEDF*, rs1136287 (c.C215T, p.Met72Thr), might be a genetic marker for AMD^38^. This amino acid substitution is located at the end of a helix domain of the PEDF protein and leads to a BsstSI restriction site^39^, suggesting it could have functional impact. Later, rs1136287 was found to be associated with AMD in the Taiwanese and Korean populations^40^,^41^, providing initial evidences to support *PEDF* as a susceptibility gene for AMD. Subsequent studies in Caucasians and other Asian cohorts, including Japanese and Chinese, did not identify a significant association between rs1136287 and AMD, but the effects of the SNP, represented by the odds ratio (OR), were variable across study cohorts^42^,^43^,^44^,^45^,^46^. As such, whether *PEDF* rs1136287 is a genuine genetic marker for AMD remains inconclusive. Also, whether there is population-specific association of this SNP with AMD needs further confirmation. Moreover, SNPs other than rs1136287, such as rs12150053, rs12948385 and rs9913583 in *PEDF* had also been reported in AMD or PCV^41^,^43^,^44^,^45^,^46^, but their associations remains inconclusive.

Since PEDF is functionally important in AMD pathogenesis and could be a new target for AMD treatment, the identification of disease-associated gene variants could provide useful targets for studying the roles of PEDF in AMD pathogenesis and pharmacogenetics. To confirm the role of *PEDF* as a candidate gene for AMD and PCV, we conducted a systematic review and meta-analysis to evaluate the associations of all reported *PEDF* SNPs with AMD and PCV. This report is about the results of the meta-analysis.

## Results

### Characteristics of eligible studies on *PEDF* in AMD and/or PCV

**Figure 1:**
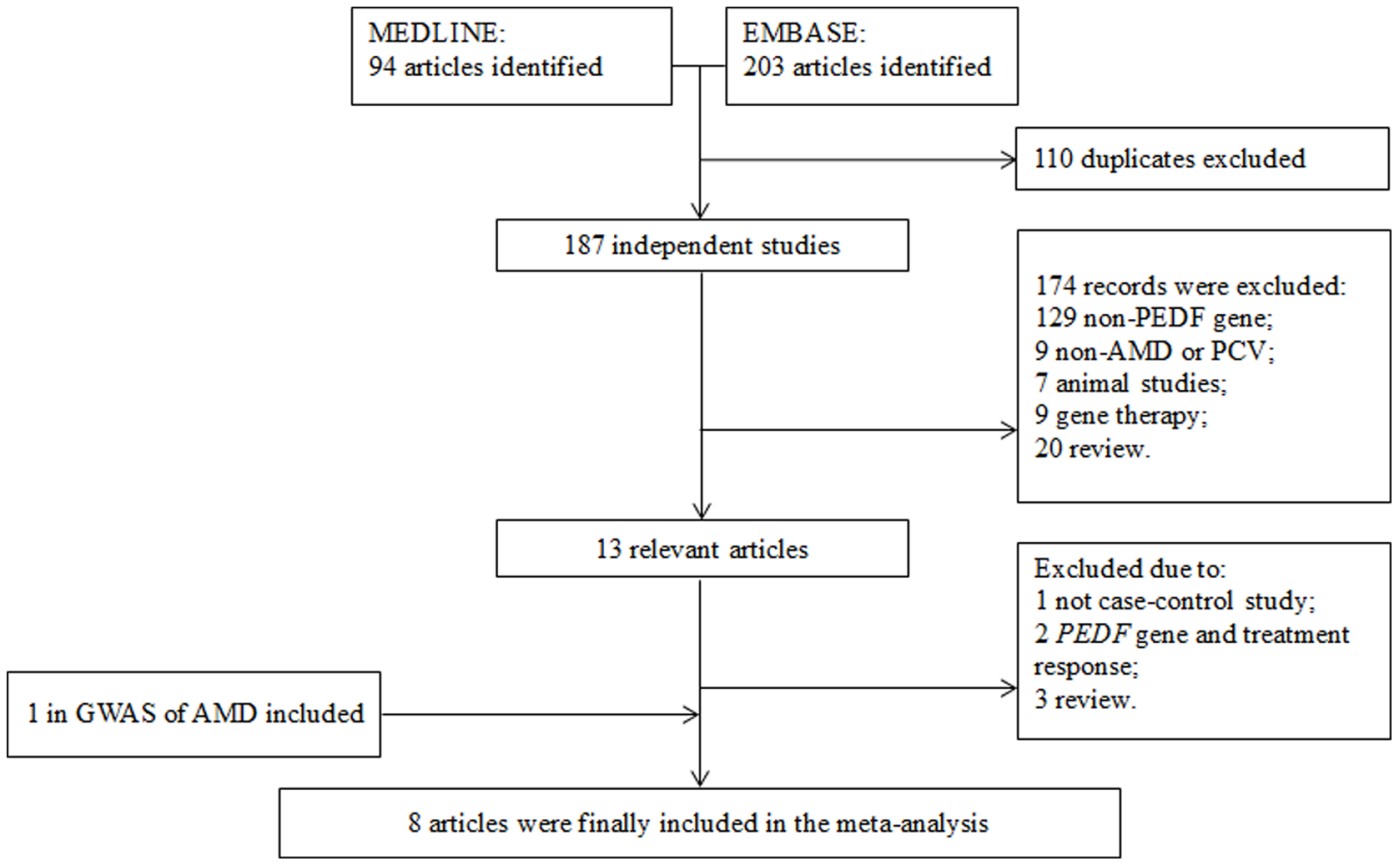
Flow diagram and results of literature review. The flow diagram depicts the screening process of retrieved articles, including the number and reason of exclusion. AMD: age-related macular degeneration; GWAS: genome-wide association study; PCV: polypoidal choroidal vasculopathy.

Figure 1 showed the study inclusion of this meta-analysis. A total of 297 articles published between January 1, 1980 and August 21, 2014 were identified in the EMBASE and MEDLINE databases. By excluding duplicated and unrelated records, we got 13 relevant reports for further assessment. However, 6 of them were excluded because 2 articles are about *PEDF* and the treatment response in AMD and PCV^47^,^48^, 1 is not a case-control study^38^, and the other 3 are reviews^49^,^50^,^51^. By searching the reference lists of relevant studies and genome-wide association studies (GWAS) of AMD, we identified another 2 relevant articles^52^,^53^. However, one GWA study did not provide genotype or allele data in controls and was excluded from further analysis^52^. Therefore, a total of 8 case-control studies were finally included for the meta-analysis^40^,^41^,^42^,^43^,^44^,^45^,^46^,^53^, involving 2284 AMD cases versus 3416 controls, and 317 PCV cases versus 371 controls. The main characteristics of the included studies were summarized in Table 1. The sample sizes of AMD groups ranged from 109 to 893, PCV groups 140 to 177, and control groups 90 to 2199. The mean age ranged from 70.9 to 78.6 years in the AMD groups, 65 to 73 years in the PCV groups, and 44 to 77.4 years in the control groups. The gender ratios (male/female) varied from 0.51 to 3.46 in the AMD groups, 1.95 to 3.38 in the PCV groups, and 0.80 to 1.63 in the control groups. The subjects in 6 studies were Asians and 2 were Caucasians. Of the six studies on nAMD, 2 involved both nAMD and PCV, and 1 involved combined nAMD, non-neovascular AMD and PCV.

### Risk of bias assessment in eligible studies

As shown in Table 2, risk of ascertainment bias in the diagnostic criteria for AMD and PCV was present in 3 studies, in which subjects were not defined based on fundus fluorescein angiography (FFA) and indocyanine green angiography (ICGA)^40^,^41^,^53^. One paper did not mention the ascertainment of control subjects^41^. Risk of population stratification presented in 1 study, which used spouses and friends as control subjects^53^. There was risk of confounding bias in 5 studies, in which results were not adjusted for confounding variables^40^,^41^,^45^,^46^,^53^. All the studies reported Hardy-Weinberg equilibrium (HWE) in controls.

### Meta-analysis of *PEDF* polymorphisms in AMD

Totally 9 SNPs had been tested in AMD and/or PCV in the literature. However, only 4 SNPs (rs1136287, rs12150053, rs12948385 and rs9913583) in AMD and 1 SNP (rs1136287) in PCV were reported in at least two studies and thus eligible for meta-analysis. Summary of the allelic associations of these four *PEDF* polymorphisms are shown in Table 3. The other 5 SNPs, i.e., rs11658342, rs1894286, rs2269344, rs9889773 and rs3786136, appeared in single report but showed no association with AMD^45^,^54^. We also searched for SNP IDs that were merged with *PEDF* SNPs and found that rs1804144, rs3199567, rs16951641, rs17352972, rs17845405, rs17858264 and rs58553017 have been merged into rs1136287 in the dbSNP database. However, none of these SNP IDs except rs1136287 was adopted in the studies.

**Figure 2:**
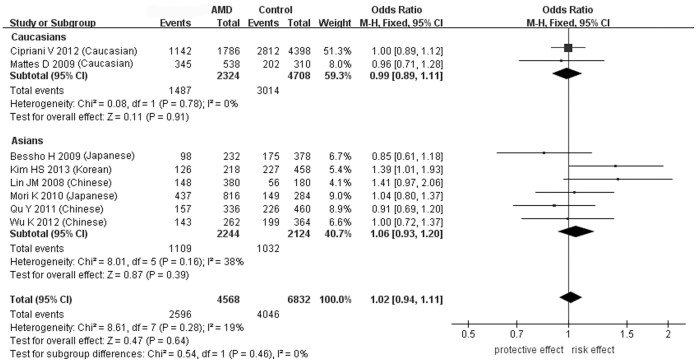
Forest plot of rs1136287(T) in AMD in allelic model. Squares indicate study-specific odds ratios (ORs). The size of the box is proportional to the weight of the study. Horizontal lines indicate 95% confidence intervals (CI). A diamond indicates the summary OR with its corresponding 95% CI. AMD: age-related macular degeneration. The cases in the study of Lin et al. included both nAMD and atrophic AMD.

SNP rs1136287 is the most-investigated SNP in AMD, with a total of 2284 cases and 3416 controls included for the meta-analysis^40^,^41^,^42^,^43^,^44^,^45^,^46^,^53^. The pooled results showed no statistically significant association between rs1136287 and all forms of AMD (Table 3 and Fig. 2). In the allelic model, the odds ratio for the risk allele T was 1.02 (95% confidence intervals (CI): 0.94–1.11, *P* = 0.64, *I^2^* = 19%). Also, the associations were not significant under the dominant, recessive, heterozygous and homozygous models (Supplementary Table S1). In the sensitivity analysis, we excluded geographic atrophy and non-neovascular AMD from the meta-analysis^40^,^44^,^53^ because *PEDF* was reportedly associated only with CNV^22^,^23^,^24^. Still, the pooled allelic OR was not statistically significant (OR = 1.12, 95% CI: 0.87–1.43, *P* = 0.38, *I^2^* = 71%)^40^,^41^,^42^,^43^,^45^,^46^. Quality assessment showed that the Korean cohort was of higher risk of inducing bias than the other cohorts due to insufficient data of clear diagnostic criteria and confounding factors (e.g. gender, age, and smoking status)^41^. Therefore, we excluded the Korean cohort in the sensitivity analysis. Still the pooled allelic OR was non-significant (OR = 1.00, 95% CI: 0.92–1.09, *P* = 0.97, *I^2^* = 0%). We also removed the Korean and Caucasian cohorts to include only Asian studies of high quality, but there was no significant change (OR = 1.01, 95% CI: 0.88–1.15, *P* = 0.93, *I^2^* = 15%). In the subgroup analysis by ethnicity, still no significant association was detected in Caucasians (OR = 0.99, 95% CI: 0.89–1.11, *P* = 0.91, *I^2^* = 0%), Japanese (OR = 0.96, 95% CI: 0.78–1.18, *P* = 0.71, *I^2^* = 0%), or Chinese (OR = 1.04, 95% CI: 0.87–1.25, *P* = 0.66, *I^2^* = 43%; Supplementary Table S2).

**Figure 3:**
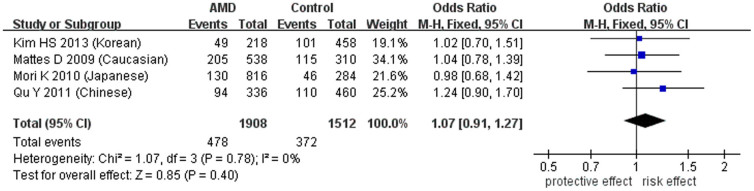
Forest plot of rs12150053(C) in AMD in allelic model. Squares indicate study-specific odds ratios (ORs). The size of the box is proportional to the weight of the study. Horizontal lines indicate 95% confidence intervals (CI). A diamond indicates the summary OR with its corresponding 95% CI. AMD: age-related macular degeneration.

**Figure 4:**
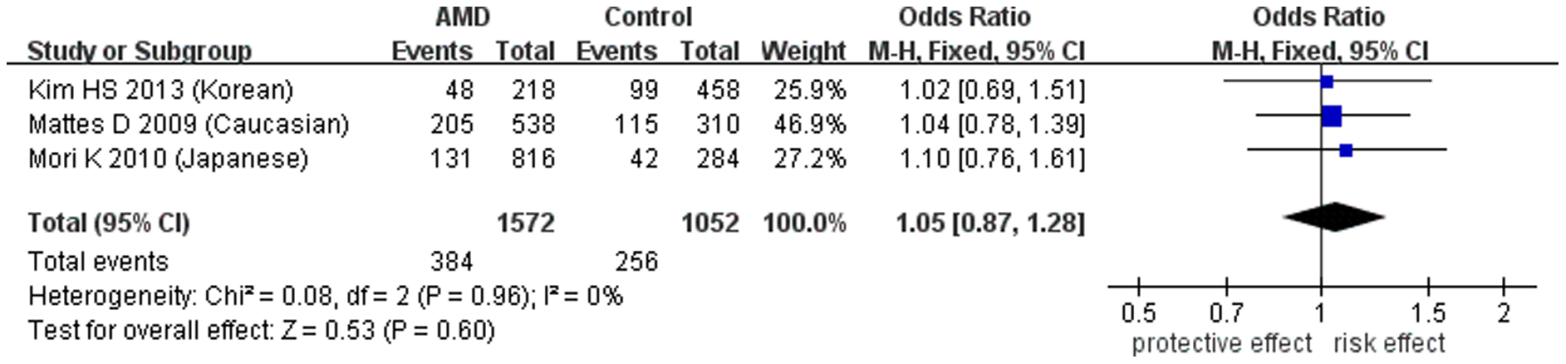
Forest plot of rs12948385(A) in AMD in allelic model. Squares indicate study-specific odds ratios (ORs). The size of the box is proportional to the weight of the study. Horizontal lines indicate 95% confidence intervals (CI). A diamond indicates the summary OR with its corresponding 95% CI. AMD: age-related macular degeneration.

**Figure 5:**
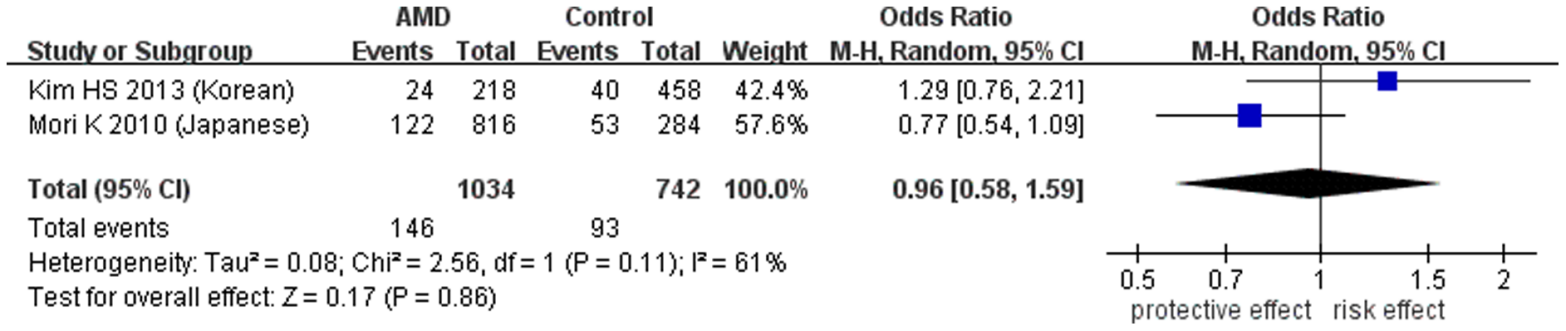
Forest plot of rs9913583(A) in AMD in allelic model. Squares indicate study-specific odds ratios (ORs). The size of the box is proportional to the weight of the study. Horizontal lines indicate 95% confidence intervals (CI). A diamond indicates the summary OR with its corresponding 95% CI. AMD: age-related macular degeneration.

Regarding the other three SNPs, rs12150053 (c.-5736T>C), rs12948385 (c.-5304G>A) and rs9913583 (c.-86C>A), which are located in the promoter region or 5′-untranslated region of *PEDF*, the pooled ORs were not statistically significant in AMD in the allelic (Table 3 and Figs. 3, 4, 5) or other genetic models (Supplementary Table S1). Leave-one-out sensitivity analysis indicated that there was no substantial change after excluding any individual study, which manifested stable results (*P* > 0.1). Also, there was no publication bias for any SNPs according to the funnel plots and Egger's test (*P* > 0.1).

### Meta-analysis of *PEDF* rs1136287 in PCV

For PCV, rs1136287 was the only SNP that was repeatedly studied. It was reported in 2 studies, involving a total of 317 cases and 371 controls^42^,^46^. Meta-analysis showed a lack of significant association between rs1136287 and PCV (OR = 0.99, 95% CI: 0.80–1.22, *P* = 0.91, *I^2^* = 25%; Table 3 and Supplementary Fig. S1). We combined all AMD and PCV studies with an attempt to increase statistical power. Still no significant association was detected (OR = 1.02, 95% CI: 0.94–1.10, *P* = 0.61, *I^2^* = 12%).

## Discussion

In this systematic review and meta-analysis, we have, for the first time, summarized the association profiles of *PEDF* in AMD and PCV, and we found no significant association between reported *PEDF* SNPs and AMD/PCV in overall samples or different ethnic subgroups. Therefore, current literatures do not support the role of *PEDF* as a susceptibility gene for AMD and PCV, despite the PEDF protein is functionally important in choroidal neovascularization.

PEDF, an antiangiogenic and neurotrophic factor, involves in the neuronal survival and differentiation in the eye and brain. The *PEDF* gene, also known as *SERPINF1*, is located on chromosomal region 17p13.1. It contains 8 exons, encoding a polypeptide of 418 amino acids. *PEDF* was considered as a disease gene for AMD because (1) the PEDF protein is one of the most effective endogenous inhibitors of angiogenesis and neovascularization^28^,^55^; (2) decreased PEDF level was found in the vitreous of AMD patients than controls, suggesting that the deficiency of PEDF in the eye could play a role in the pathogenesis of AMD^18^; (3) intravitreal injection of PEDF-expressing adenoviral vector reduced CNV size in AMD^37^; and (4) there were studies reporting significant association of *PEDF* SNPs with AMD and/or PCV.

In the studies of Lin et al., rs1136287 was found to be associated with nAMD in Taiwanese, with an odds ratio of 2.21 (95% CI: 1.43–3.42, *P* = 0.005) for the T allele, but not with atrophic AMD (OR = 0.94, 95% CI: 0.61–1.45, *P* = 0.87)^40^. Also rs1136287 was associated with nAMD in a Korean population (OR = 1.39, 95% CI: 1.01–1.93, *P* = 0.045)^41^. However, as summarized in our meta-analysis, in another 6 studies (including 2 in Caucasians^43^,^53^, 2 in Japanese^42^,^44^ and 2 in Chinese^45^,^46^) no significant allelic association was found for rs1136287, although the heterozygous genotype (CT) showed a protective effect for AMD (OR = 0.59, 95% CI: 0.36–0.95, *P* = 0.03) in the study of Qu et al^45^. So far the largest sample size for rs1136287 was reported in a GWAS involving 893 cases and 2199 controls of Caucasian origin, in which no association was found with advanced AMD (OR = 1.00, 95% CI: 0.89–1.12, *P* = 0.97)^53^. Therefore, together with the stratification analysis in our meta-analysis, we conclude that SNP rs1136287 is not a susceptibility genetic marker for AMD in Caucasians. This leads to a question that whether the association of rs1136287 with AMD is population- or disease subtype-specific. Notably, the criteria for AMD classification for the subjects in all the included studies were consistent, except the Korean cohort, in which no explicit criteria for AMD were described^44^. Thus, subtype-specific association is less likely, especially for the Taiwanese cohort. Also, in our meta-analysis, we found no significant association of rs1136287 with all forms of AMD in Caucasians or Asians, with the ORs towards different directions in different populations and small heterogeneity among studies (Fig. 2). This suggests the associations detected in the Taiwanese and Korean cohorts might be chance findings from a limited sample size, or there could be population-specific association of this SNP, confirmation of which requires replication among Taiwanese and Korean populations.

In recent years, GWAS, a hypothesis-free approach, has become a predominant method for identifying associated loci for AMD. However, findings across different GWAS had been variable^4^,^7^,^53^,^56^,^57^. This could be due to the variation in sample sizes and ethnicities, leading to variable association signals. For example, the association signal at the 4q12 gene cluster, first identified in the Japanese population^56^, was not detected in a recent large-scale AMD GWAS in Caucasians^57^, suggesting population-specific effect. Moreover, in GWAS only SNPs reaching a predefined threshold are replicated, thus some disease-associated SNPs might have been missed, resulting in false negatives. Therefore, on the one hand, results from multiple cohorts should be incorporated to validate the initial signals by methods such as meta-analysis; whereas on the other hand, methods other than GWAS should play a role in gene identification. The candidate gene approach, which is hypothesis-based, has been an important method for mapping AMD genes. For example in the complement pathway, while the *CFH* gene was identified for AMD by GWAS, the complement components 2 and 3 (*C2* and *C3*), and complement factor B (*CFB*) were identified under the hypothesis that variation in genes encoding proteins of the same pathway with CFH could be associated with AMD^58^,^59^. These genes have later been confirmed in genotyping studies^57^ or meta-analysis^60^ on AMD and PCV^61^, suggesting the importance of candidate gene analysis in the discovery of AMD genes. In our present meta-analysis, we aimed to confirm the role of an excellent candidate gene for AMD - *PEDF*, but we found no significant association of a major SNP rs1136287 with AMD or PCV. Nevertheless, our observation that no replication study has yet been available in Taiwanese and Korean populations may arouse follow-up studies on this SNP in the two populations.

Apart from rs1136287, we also found that another 3 *PEDF* SNPs, rs12150053, rs12948385 and rs9913583, were not associated with AMD. Notably among the included studies, only one involved haplotype-tagging SNPs in the association analysis^45^. In the study, 4 tagging SNPs (rs11658342, rs1894286, rs2269344 and rs9889773) with r^2^ cutoff of 1.0 and minor allele frequency (MAF) cutoff of 5%, and 3 reported SNPs (rs1136287, rs12150053 and rs12948385) were selected. The CT genotype of rs1136287 was found to be associated with AMD (OR = 0.59, 95% CI: 0.36–0.95, *P* = 0.03), although the allelic associations of all SNPs were non-significant^45^. Since a r^2^ cutoff of 1.0 is likely to limit the number of SNPs that capture the major proportion of gene information, we searched for the tagging SNPs of *PEDF* using a MAF cutoff of 5% and r^2^ cutoff of 0.8 in the HapMap Genome Browser (release No. 27, http://hapmap.ncbi.nlm.nih.gov/; accessed Oct 27, 2014), and we identified more SNPs (Supplementary Table S3). Among the included studies, one tagging SNP rs1136287 in the CHB (Han Chinese in Beijing) population was included in 3 Chinese cohorts^40^,^45^,^46^, while one tagging SNP rs9913583 in the JPT (Japanese in Tokyo) population was studied in 1 Japanese cohort^44^. Since haplotype-tagging SNP analysis is useful for identifying the responsible SNP in a genetic locus^62^, further studies using the tagging SNPs would be warranted to provide a comprehensive evaluation of *PEDF* in AMD and PCV.

Upon identifying a lack of association of the *PEDF* SNPs with AMD and PCV, we searched for possible association of *PEDF* with other ophthalmic and non-ophthalmic diseases with a view to better understand the role of *PEDF*. Previously, based on linkage analysis, Koenekoop et al. suggested *PEDF* as a causative gene for Leber's congenital amaurosis^63^. In the study of Miyake et al., *PEDF* rs12603825 was found to have marginal association with myopic CNV, while rs1136287 was found of no association with CNV in Japanese myopic patients^64^. A meta-analysis is not possible because only one article was found for each SNP. Of note, *PEDF* was evaluated in diabetic retinopathy in three studies^39^,^65^,^66^. However, three SNPs, rs1136287, rs12150053 and rs12948385, showed no significant association with DR by meta-analysis (Supplementary Fig. S2).

The current systematic review and meta-analysis is an overview of published genetic reports on *PEDF* in AMD and PCV. Our study revealed several limitations in this topic. First, AMD and PCV are multifactorial diseases involving both environmental and genetic factors. Our results were based on unadjusted assessment and indicated discrepancy even in the same ethnicity, suggesting environmental factors may be involved. Thus, a more rigorous analysis should be performed by stratifying other risk factors if data was available. Second, we found a limited number of studies for this meta-analysis and most cohorts were Asians. In particular, only 2 studies with relatively small sample sizes reported the association of *PEDF* with PCV. Therefore, a comprehensive association of *PEDF* with AMD and PCV should be elucidated in more study cohorts. Also, no replication study was available in the Taiwanese and Korean populations, in which significant association of rs1136287 with AMD was detected. Therefore, further studies should be performed in these 2 populations. Third, significant heterogeneity was detected for some SNPs (e.g., rs9913583 in AMD), thus the random-effect model was applied to yield more conservative odds ratio. Fourth, some studies got low score of quality assessment^40^,^41^ or did not give the classification of AMD^44^, which may cause imprecise results when AMD or PCV was evaluated separately. However, since our meta-analysis showed no association of *PEDF* SNPs with either AMD or PCV, the lack of classification in the initial studies would have no major impact to the final conclusion.

In conclusion, this systematic review and meta-analysis has, for the first time, provided an overview of reported *PEDF* SNPs in AMD and PCV, and the results suggest that *PEDF* is not a major susceptibility gene for the diseases in the overall population. However, further studies should be warranted to confirm the association of *PEDF* SNP rs1136287 with AMD in specific populations such as Taiwanese and Korean. Moreover, since the pooled sample size for PCV was small, further studies of *PEDF* in large PCV samples are warranted.

## Methods

### Literature search

Literature search was done in the MEDLINE and EMBASE databases for genetic studies on *PEDF* in AMD and/or PCV. We used MeSH terms and free words: (Pigment epithelium-derived factor or PEDF or SERPINF1 or serpin peptidase inhibitor, clade F or OI6 or OI12 or EPC-1 or PIG35) and (age-related macular degeneration or AMD or ARMD or age-related macular disease or age-related maculopathy or ARM or PCV or polypoidal choroidal vasculopathy). All related articles published before August 21, 2014 were retrieved without language restriction. To find more papers, we manually screened the reference lists of all eligible articles. Moreover, to maximize the usable data we also searched all reported genome-wide association studies of AMD including their supplementary materials. Details of search strategy were illuminated in Supplementary Table S4.

### Inclusion and exclusion criteria

A study was included if it fulfills all the following criteria: (1) case-control study, cohort study or population-based study investigating the association between *PEDF* and AMD and/or PCV; (2) data of genotype and/or allele counts or frequencies were presented in the papers; (3) unrelated control subjects were free of AMD or PCV; (4) for articles published by the same group of authors on the same gene or markers, only the latest study or the one with the largest sample size was included. Animal researches, case report, reviews, conference report, editorial comment and reports without sufficient data were excluded (Supplementary Table S5).

### Literature review and data extraction

Two reviewers (L.M. and S.M.T.) independently reviewed and extracted data from studies on the association between *PEDF* SNPs and AMD/PCV. Any discrepancies were resolved by another two reviewers (S.S.R. and L.J.C.) after thorough discussion. The following information was extracted from each record: the name of first author, publication year, ethnicity of the study population, study design, sample size, disease subtype, gender composition, mean age, allele and genotype distribution in cases and controls, Hardy-Weinberg equilibrium (HWE) test results in controls. The records were combined into one group if listed allele and/or genotype distribution was stratified by AMD classification^40^.

### Risk of bias assessment

The reviewers appraised the qualities of retrieved records by a modified risk-of-bias score for genetic association studies, based on traditional epidemiologic and genetic considerations (Supplementary Table S6)^67^,^68^,^69^. The assessment consists of 3 domains: (1) information bias: evaluation of diagnostic criteria for AMD, PCV and controls; (2) confounding bias: assessment of population bias and other confounding variables; (3) consideration of HWE in individual study. Each domain contains 3 answers: yes, no or unclear, which presents low risk of bias, high risk of bias and unclear if the included study was assessed based on insufficient information.

### Statistical analysis

Meta-analysis for each polymorphism was performed if it had been reported in ≥2 studies or cohorts. The association was assessed using different genetic models, including allelic, dominant, recessive, heterozygous and homozygous models. Pooled odds ratios and 95% confidence intervals of each SNP were estimated for the strength of association, using the fixed-effect (*I^2^* ≤ 50%) or random-effect (*I^2^* > 50%) model based on the heterogeneity test^70^. The *I^2^* test was used to assess heterogeneity among studies. The *I^2^* value was explained as of no (0–25%), low (25–50%), moderate (50–75%) or high heterogeneity (75–100%)^71^.

Sensitivity analysis was carried out to examine the influence by removing one study each time^69^. The potential publication bias was evaluated with funnel plots and the Egger's test^72^,^73^. When the p value of the Egger test was <0.05, publication bias was expected to exist. Statistical analyses were conducted using the software Review Manager (RevMan, version 5.2, The Cochrane Collaboration, Copenhagen, Denmark), and Egger's test was performed in R (version 2.15.0, http://cran.r-project.org/). A pooled *P* value of less than 0.05 was considered statistically significant.

## Author Contributions

L.M., S.S.R. and L.J.C. conceived and participated in its design and searched databases. L.M. and S.M.T. reviewed and extracted data. L.M. and S.S.R. carried out the statistical analysis and interpretation of data. L.M. and L.J.C. drafted and revised the article. H.Y.C., A.L.Y., G.K. and C.P.P. read and approved the final manuscript.

## Supplementary Material

Supplementary Information supplementary material

## Figures and Tables

**Table 1:** Characteristics of studies included in the meta-analysis

Study	Ethnicity	Sample size		Gender (male/female)		Age (mean ± SD, years)		Subtype
		Case	Control	Case	Control	Case	Control	
**AMD**								
Bessho H 2009	Japanese	116	189	90/26	113/76	75.0 ± 7.2	72.0 ± 5.8	nAMD
Kim HS 2013	Korean	109	229	NA	NA	NA	NA	nAMD
Lin JM 2008	Chinese	190	90	108/82	49/41	71.1 ± 7.5	71.5 ± 6.9	nAMD and atrophic AMD
Mattes D 2009	Caucasian	269	155	91/178	69/86	71.4 ± 6.4	71.5 ± 6.9	nAMD
Mori K 2010	Japanese	408	142	313/95	74/68	78.4 ± 7.0	77.4 ± 6.5	nAMD, PCV and nnAMD^a^
Qu Y 2011	Chinese	168	230	95/73	134/96	71.9 ± 8.5	68.4 ± 9.8	nAMD
Wu K 2012	Chinese	131	182	84/57	113/69	70.9 ± 11.3	74.4 ± 9.6	nAMD
Cipriani V 2012^b^	Caucasian	893	2199	399/494	1082/1117	78.6 ± 7.5	44–45^b^	Advanced AMD
**PCV**								
Bessho H 2009	Japanese	140	189	108/32	113/76	73.0 ± 6.9	72.0 ± 5.8	PCV
Wu K 2012	Chinese	177	182	117/60	113/69	65.0 ± 8.45	74.4 ± 9.6	PCV

^a^Combined subtypes.

^b^Data extracted from a genome-wide association study; for the control subjects only age range was given in the study.

nAMD: neovascular AMD; nnAMD: non-neovascular AMD; PCV: polypoidal choroidal vasculopathy; NA: not available from the initial report; SD: standard deviation.

**Table 2:** Risk of bias assessment of included studies in the meta-analysis

Study	Ascertainment of AMD and PCV	Ascertainment of controls	Population stratification	Confounding bias	HWE in controls
Bessho H 2009	Yes	Yes	Yes	Yes	Yes
Kim HS 2013	Unclear	Unclear	Yes	No	Yes
Lin JM 2008	No	Yes	Yes	No	Yes
Mattes D 2009	Yes	Yes	Yes	Yes	Yes
Mori K 2010	Yes	Yes	Yes	Yes	Yes
Qu Y 2011	Yes	Yes	Yes	No	Yes
Wu K 2012	Yes	Yes	Yes	No	Yes
Cipriani V 2012	No	Yes	No	No	Yes

Yes, no and unclear presented low risk of bias, high risk of bias and insufficient information, respectively.

AMD: age-related macular degeneration; PCV: polypoidal choroidal vasculopathy; HWE: Hardy-Weinberg equilibrium.

**Table 3:** Meta-analyses of allelic association of *PEDF* polymorphisms with AMD or PCV

Polymorphism	Ethnicity	Alleles	Number of cohorts	Sample size (case/control)	OR (95% CI)	Z score	P-value	*I^2^* (%)
rs1136287	All ancestries	T vs C	8	2284/3416	1.02 (0.94–1.11)	0.47	0.64	19
(c.C215T, p.Met72Thr)	Asian		6	1122/1062	1.06 (0.93–1.20)	0.87	0.39	38
	Caucasian		2	1162/2354	0.99 (0.89–1.11)	0.11	0.91	0
	Asian PCV^a^	T vs C	2	317/371	0.99 (0.80–1.22)	0.11	0.91	25
rs12150053	All ancestries	C vs T	4	954/756	1.07 (0.91–1.27)	0.85	0.40	0
	Asian		3	685/601	1.09 (0.89–1.34)	0.83	0.41	0
rs12948385	All ancestries	A vs G	3	786/526	1.05 (0.87–1.28)	0.53	0.60	0
	Asian		2	517/371	1.06 (0.81–1.39)	0.45	0.65	0
rs9913583	Asian	A vs C	2	517/371	0.96 (0.58–1.59)	0.17	0.86	61

^a^Only one SNP, rs1136287, was reported in PCV; the others were for AMD.

AMD: age-related macular degeneration; PCV: polypoidal choroidal vasculopathy; OR: odds ratio; CI: confidence intervals.
