# Supplementary material for: Association of PEDF polymorphisms with age-related macular degeneration and polypoidal choroidal vasculopathy: a systematic review and meta-analysis
Source: Sci Rep. 2015 Mar 30;5:9497. doi: 10.1038/srep09497 (PMC4377572; doi:10.1038/srep09497)
Supplement: Supplementary Information — supplementary material [file srep09497-s1.pdf]

Association of *PEDF* polymorphisms with age-related macular degeneration and polypoidal choroidal vasculopathy: a systematic review and meta-analysis

Li Ma,[1] Shu Min Tang,[1] Shi Song Rong,[1] Haoyu Chen,[3] Alvin L. Young,[1,2] Govindasamy Kumaramanickavel,[4] Chi Pui Pang,[1,2,3] Li Jia Chen.[1,2*]

[1]Department of Ophthalmology and Visual Sciences, The Chinese University of Hong Kong, Hong Kong, China, [2]Department of Ophthalmology and Visual Sciences, Prince of Wales hospital, Hong Kong, China, [3]The Shantou University/Chinese University of Hong Kong Joint Shantou International Eye Center, Shantou, Guangdong Province, China, [4]Narayana Nethralaya, Health City, Bangalore, India.

Correspondence:

Dr Li Jia Chen

Department of Ophthalmology and Visual Sciences, The Chinese University of Hong Kong, Prince of Wales Hospital, Shatin, New Territory, Hong Kong.

Tel: +852 39435810 Fax: +852 27159490 E-mail: lijia_chen@cuhk.edu.hk

Table S1. Meta-analyses on the association of *PEDF* SNPs with AMD in different genetic models

SNP	Ethnicity	No. of cohorts	Sample size (case/control)	Genetic model		OR (95% CI)	*P*	I^2 (%)
rs1136287	All ancestries	8	2284/3416	Dominant	(TT+TC vs. CC)	1.00 (0.87-1.15)	0.96	13
				Recessive	(TT vs. TC+ CC)	1.05 (0.94-1.19)	0.39	0
				Heterozygous	(TC vs. CC)	0.95 (0.81-1.11)	0.54	2
				Homozygous	(TT vs. CC)	1.04 (0.88-1.24)	0.61	16
rs12150053	All ancestries	4	954/756	Dominant	(CC+CT vs. TT)	1.10 (0.89-1.36)	0.37	0
				Recessive	(CC vs. CT+ TT)	1.05 (0.72-1.52)	0.81	0
				Heterozygous	(CT vs. TT)	1.10 (0.88-1.37)	0.41	0
				Homozygous	(CC vs. TT)	1.11 (0.75-1.65)	0.59	0
rs12948385	All ancestries	3	786/526	Dominant	(AA+ AG vs. GG)	1.08 (0.84-1.38)	0.54	0
				Recessive	(AA vs. AG+ GG)	1.01 (0.65-1.57)	0.97	0
				Heterozygous	(AG vs. GG)	1.08 (0.83-1.41)	0.55	0
				Homozygous	(AA vs. GG)	1.08 (0.67-1.72)	0.76	0
rs9913583	Asians	2	517/371	Dominant	(AA+AC vs. CC)	0.86 (0.61-1.21)	0.38	34
				Recessive	(AA vs. AC+ CC)	1.61 (0.17-15.66)	0.68	70
				Heterozygous	(AC vs. CC)	0.84 (0.59-1.19)	0.33	0
				Homozygous	(AA vs. CC)	1.57 (0.15-16.57)	0.71	72

AMD: age-related macular degeneration; OR: odds ratio; CI: confidence intervals; SNP: single nucleotide polymorphism.

Table S2. Meta-analyses on the allelic association of *PEDF* rs1136287(T) with AMD in different subgroups

Ethnicity	No. of cohorts	Sample size (case/control)	OR (95% CI)	*P*	I^2 (%)
Caucasian	2	1162/2354	0.99 (0.89-1.11)	0.91	0
Japanese	2	524/331	0.96 (0.78-1.18)	0.71	0
Chinese	3	489/502	1.04 (0.87-1.25)	0.66	43
All cohorts except Korean	7	2175/3187	1.00 (0.92-1.09)	0.97	0
All Asian cohorts except Korean	5	1013/833	1.01 (0.88-1.15)	0.93	15

AMD: age-related macular degeneration; OR: odds ratio; CI: confidence intervals.

Table S3. Haplotype-tagging SNPs of *PEDF* in different populations

Tag SNPs	Captured SNPs
**CEU**	
rs4274475	rs4274475, rs2071021, rs12453107, rs9889773, rs8074840
rs5008943	rs4274474, rs5008943, rs6828, rs2269344, rs4511575
rs12450371	rs12450371, rs6502953, rs1136287
rs11658342	rs11658342
rs12603825	rs12603825
**CHB**	
rs5008943	rs4511575, rs6828, rs4274474, rs5008943, rs2269344
rs1894286	rs3891224, rs12103559, rs1894286
rs2071021	rs4274475, rs8074840, rs2071021
rs1136287	rs1136287, rs6502953, rs12450371
rs9889773	rs9889773, rs12453107
rs12325945	rs12325945
rs12603825	rs12603825
rs11658342	rs11658342
**JPT**	
rs5008943	rs2269344, rs5008943, rs4274474, rs6828, rs4511575
rs2071021	rs4274475, rs8074840, rs2071021
rs12103559	rs3891224, rs12103559, rs1894286
rs12450371	rs6502953, rs1136287, rs12450371
rs12453107	rs12453107, rs9889773
rs9913583	rs9913583, rs12325945
rs12603825	rs12603825
rs11658342	rs11658342
rs12603486	rs12603486

SNP: single nucleotide polymorphism; CEU: Utah residents with Northern and Western European ancestry from the CEPH collection; CHB: Han Chinese in Beijing, China; JPT: Japanese in Tokyo, Japan.

Table S4. Search strategy for EMBASE and MEDLINE (Ovid):

Code	Key words
1	PEDF
2	Pigment epithelium-derived factor
3	SERPINF1
4	Serpin peptidase inhibitor, clade F
5	OI6
6	OI12
7	EPC-1
8	PIG35
9	AMD
10	Age-related macular degeneration
11	ARMD
12	Age-related macular disease
13	Age-related maculopathy
14	ARM
15	PCV
16	Polypoidal choroidal vasculopathy
17	(1 OR 2 OR 3 OR 4 OR 5 OR 6 OR 7 OR 8)
18	(9 OR 10 OR 11 OR 12 OR 13 OR 14)
19	(15 OR 16)
20	17 AND (18 OR 19)

Table S5. Lists of included/excluded studies with reasons

No.	Year	First Author	Journal	Title	Reason for inclusion/exclusion
Included studies					
1	2008	Lin JM	American Journal of Ophthalmology	Pigment Epithelium-Derived Factor Gene Met72Thr Polymorphism Is Associated With Increased Risk of Wet Age-related Macular Degeneration..	Eligibility
2	2009	Bessho H	Molecular Vision	Coding variant Met72Thr in the PEDF gene and risk of neovascular age-related macular degeneration and polypoidal choroidal vasculopathy.	Eligibility
3	2009	Mattes D	Molecular Vision	Analysis of three pigment epithelium-derived factor gene polymorphisms in patients with exudative age-related macular degeneration.	Eligibility
4	2010	Mori K	Ophthalmology	Phenotype and Genotype Characteristics of Age-related Macular Degeneration in a Japanese Population.	Eligibility
5	2011	Qu Y	Current Eye Research	Pigment epithelium-derived factor gene polymorphisms in exudative age-related degeneration in a Chinese cohort.	Eligibility
6	2012	Cipriani V	Human Molecular Genetics	Genome-wide association study of age-related macular degeneration identifies associated variants in the TNXB–FKBPL–NOTCH4 region of chromosome 6p21.3.	GWAS, eligibility
7	2012	Wu K	Current Eye Research	Lack of association with PEDF Met72Thr variant in neovascular age-related macular degeneration and polypoidal choroidal vasculopathy in a Han Chinese population.	Eligibility
8	2013	Kim HS	Genes and Genomics	Genetic association of VEGF and PEDF polymorphisms with age-related macular degeneration in Korean.	Eligibility
Excluded studies					
1	2001	Chader GJ	Proceedings of the National Academy of Sciences of the United States of America	PEDF: Raising both hopes and questions in controlling angiogenesis	Non-*PEDF* gene

2	2001	Ohno-Matsui K	Journal of Cellular Physiology	Novel mechanism for age-related macular degeneration: An equilibrium shift between the angiogenesis factors VEGF and PEDF.	Review
3	2001	Rasmussen H	Human gene therapy	Clinical protocol. An open-label, phase I, single administration, dose-escalation study of ADGVPEDF.11D (ADPEDF) in neovascular age-related macular degeneration (AMD).	Non-*PEDF* gene
4	2001	Rasmussen HS	Drug Discovery Today	Looking into anti-angiogenic gene therapies for disorders of the eye.	Gene therapy
5	2002	Auricchio A	Molecular Therapy	Inhibition of retinal neovascularization by intraocular viral-mediated delivery of anti-angiogenic agents.	Non-*PEDF* gene
6	2002	Holekamp NM	American Journal of Ophthalmology	Pigment epithelium-derived factor is deficient in the vitreous of patients with choroidal neovascularization due to age-related macular degeneration.	Non-*PEDF* gene
7	2002	Raisler BJ	Proceedings of the National Academy of Sciences of the United States of America	Adeno-associated virus type-2 expression of pigmented epithelium-derived factor or Kringles 1-3 of angiostatin reduce retinal neovascularization.	Animal study
8	2002	Semkova I	Proceedings of the National Academy of Sciences of the United States of America	Autologous transplantation of genetically modified iris pigment epithelial cells: A promising concept for the treatment of age-related macular degeneration and other disorders of the eye.	Animal study
9	2003	Ambati J	Survey of Ophthalmology	Age-related macular degeneration: Etiology, pathogenesis, and therapeutic strategies.	Review
10	2003	Bainbridge JWB	Clinical Science	Gene therapy for ocular angiogenesis.	Non-*PEDF* gene
11	2003	Bonnel S	Experimental Gerontology	The aging of the retina.	Non-AMD or PCV
12	2003	Campochiaro PA	Oncogene	Ocular neovascularization: A valuable model system.	Non-*PEDF* gene
13	2003	Das A	Progress in Retinal and Eye Research	Retinal and choroidal angiogenesis: Pathophysiology and strategies for inhibition.	Non-AMD or PCV
14	2003	Francis PJ	Ophthalmology Clinics of North America	Gene therapy and control of angiogenesis.	Gene therapy
15	2003	Gehlbach P	Gene Therapy	Periocular injection of an adenoviral vector encoding pigment epithelium-derived factor inhibits choroidal neovascularization.	Non-*PEDF* gene

16	2003	Holz FG	Current developments and perspectives	Pharmacological therapy for age-related macular degeneration.	Non-*PEDF* gene
17	2003	Hunt DWC	IDrugs	Status of therapies in development for the treatment of age-related macular degeneration.	Non-*PEDF* gene
18	2003	Klein ML	Ophthalmology Clinics of North America	Genetics of age-related macular degeneration.	Review
19	2003	Mechoulam H	American Journal of PharmacoGenomics	Retinopathy of prematurity: Molecular pathology and therapeutic strategies.	Non-AMD or PCV
20	2003	Miller DW	Ophthalmologe	The molecular mechanisms of neovascular age-related macular degeneration.	Review
21	2003	Ohno-Matsui K	Nippon Ganka Gakkai Zasshi - Acta Societatis Ophthalmologicae Japonicae	Molecular mechanism for choroidal neovascularization in age-related macular degeneration.	Review
22	2003	Ohno-Matsui K	Biochemical and Biophysical Research Communications	Vascular endothelial growth factor upregulates pigment epithelium-derived factor expression via VEGFR-1 in human retinal pigment epithelial cells.	Non-*PEDF* gene
23	2003	Reich SJ	Current Opinion in Genetics and Development	Gene therapy for ocular neovascularization: A cure in sight.	Gene therapy
24	2003	Tratsk KS	Graefe's Archive for Clinical and Experimental Ophthalmology	UV irradiation causes multiple cellular changes in cultured human retinal pigment epithelium cells.	Non-*PEDF* gene
25	2003	Witmer AN	Progress in Retinal and Eye Research	Vascular endothelial growth factors and angiogenesis in eye disease.	Review
26	2004	Campochiaro PA	Expert Opinion on Biological Therapy	Ocular neovascularisation and excessive vascular permeability.	Non-*PEDF* gene
27	2004	Comer GM	Drugs and Aging	Current and future treatment options for nonexudative and exudative age-related macular degeneration.	Non-*PEDF* gene
28	2004	Jiang YL	Japanese Journal of Ophthalmology	Ionizing Radiation Induces a p53-dependent Apoptotic Mechanism in ARPE-19 Cells.	Non-AMD or PCV
29	2004	Konerding MA	Expert Opinion on Therapeutic Targets	Ocular angiogenesis: Translating preclinical indications to successful clinical development.	Non-*PEDF* gene

30	2004	Martin G	Graefe's Archive for Clinical and Experimental Ophthalmology	Differential expression of angioregulatory factors in normal and CNV-derived human retinal pigment epithelium.	Non-*PEDF* gene
31	2004	Matsuoka M	British Journal of Ophthalmology	Expression of pigment epithelium derived factor and vascular endothelial growth factor in choroidal neovascular neovascular membranes and polypoidal choroidal vasculopathy.	Non-*PEDF* gene
32	2004	McFarland TJ	Expert Opinion on Biological Therapy	Gene therapy for proliferative ocular diseases.	Gene therapy
33	2004	Nowak JZ	Klinika oczna	Neovascularization in ocular tissues: mechanisms and role of proangiogenic and antiangiogenic factors.	Review
34	2004	Rolling F	Gene Therapy	Recombinant AAV-mediated gene transfer to the retina: Gene therapy perspectives.	Non-*PEDF* gene
35	2004	Rowe-Rendleman C	Brain Research Bulletin	Possible therapy for age-related macular degeneration using human telomerase.	Non-*PEDF* gene
36	2004	Schlingemann RO	Graefe's Archive for Clinical and Experimental Ophthalmology	Role of growth factors and the wound healing response in age-related macular degeneration.	Non-*PEDF* gene
37	2005	Auricchio A	Current Gene Therapy	Adeno-associated viral vectors for retinal gene transfer and treatment of retinal diseases.	Non-*PEDF* gene
38	2005	Comer GM	Expert Opinion on Emerging Drugs	Future pharmacological treatment options for nonexudative and exudative age-related macular degeneration.	Non-*PEDF* gene
39	2005	Higgins RD	Current Genomics	Ocular neovascularization: Genomic implications.	Non-*PEDF* gene
40	2005	Hosomichi J	Biochemical and Biophysical Research Communications	Involvement of the collagen I-binding motif in the anti-angiogenic activity of pigment epithelium-derived factor.	Non-AMD or PCV
41	2005	Kijlstra A	Ocular immunology and inflammation	Immunological factors in the pathogenesis and treatment of age-related macular degeneration.	Non-*PEDF* gene
42	2005	Kobierzycka A	Diabetologia Doswiadczalna i Kliniczna	Pigment epithelium-derived factor (PEDF) as a potent inhibitor of angiogenesis in diabetes.	Non-AMD or PCV
43	2005	Ng EWM	Canadian Journal of Ophthalmology	Targeting angiogenesis, the underlying disorder in neovascular age-related macular degeneration.	Non-*PEDF* gene
44	2005	Pulido JS	Canadian Journal of Ophthalmology	Rheopheresis for age-related macular degeneration: Clinical results and putative mechanism of action.	Non-*PEDF* gene

45	2005	Sun JK	International Ophthalmology Clinics	Medical treatment of choroidal neovascularization secondary to age-related macular degeneration.	Non-*PEDF* gene
46	2005	Wei L	Retina (Philadelphia, Pa)	Adenovector pigment epithelium-derived factor (AdPEDF) delivery for wet age-related macular degeneration.	Non-*PEDF* gene
47	2005	Wenzel A	Progress in Retinal and Eye Research	Molecular mechanisms of light-induced photoreceptor apoptosis and neuroprotection for retinal degeneration.	Non-*PEDF* gene
48	2005	Yamagishi S	Medical Hypotheses	Met72Thr polymorphism of pigment epithelium-derived factor gene and susceptibility to age-related macular degeneration.	Not case-control study
49	2005	Ye C	Chinese Ophthalmic Research	Research advance in neovascularization factor-pigment epithelium-derived factor.	Review
50	2005	Yoshida T	Journal of Clinical Investigation	The potential role of amyloid beta in the pathogenesis of age-related macular degeneration.	Non-*PEDF* gene
51	2005	Zou YH	International Journal of Ophthalmology	Pharmacological therapy in age-related macular degeneration (AMD).	Non-*PEDF* gene
52	2006	An E	Journal of Proteome Research	Secreted proteome profiling in human RPE cell cultures derived from donors with age related macular degeneration and age matched healthy donors.	Non-*PEDF* gene
53	2006	Augustin AJ	European Journal of Ophthalmology	Verteporfin therapy and triamcinolone acetonide: Convergent modes of action for treatment of neovascular age-related macular degeneration.	Non-*PEDF* gene
54	2006	Bakri SJ	Expert Opinion on Investigational Drugs	Anecortave acetate.	Non-AMD or PCV
55	2006	Bhutto IA	Experimental Eye Research	Pigment epithelium-derived factor (PEDF) and vascular endothelial growth factor (VEGF) in aged human choroid and eyes with age-related macular degeneration.	Non-*PEDF* gene
56	2006	Campochiaro PA	Human Gene Therapy	Adenoviral vector-delivered pigment epithelium-derived factor for neovascular age-related macular degeneration: Results of a phase I clinical trial.	Non-*PEDF* gene
57	2006	Eichler W	Current Pharmaceutical Design	Antineovascular agents in the treatment of eye diseases.	Non-*PEDF* gene
58	2006	Eter N	BioDrugs	New pharmacologic approaches to therapy for age-related macular degeneration.	Non-*PEDF* gene
59	2006	Feng X	International Journal of Ophthalmology	PEDF and neovascularization disease.	Review

60	2006	Liu Y	Chinese Ophthalmic Research	Research progress in gene transfer of PEDF for treatment of retinal and choroidal neovascular disease.	Non-*PEDF* gene
61	2006	Michels S	Expert opinion on investigational drugs	Promising new treatments for neovascular age-related macular degeneration.	Non-*PEDF* gene
62	2006	Nowak JZ	Pharmacological Reports	Age-related macular degeneration (AMD): Pathogenesis and therapy.	Review
63	2006	Patricia Becerra S	Experimental Eye Research	Focus on molecules: Pigment epithelium-derived factor (PEDF).	Review
64	2006	Semkova I	The FASEB journal : official publication of the Federation of American Societies for Experimental Biology	Overexpression of FasL in retinal pigment epithelial cells reduces choroidal neovascularization.	Animal study
65	2006	Steinle JJ	Experimental Eye Research	Cervical sympathectomy regulates expression of key angiogenic factors in the rat choroid.	Animal study
66	2006	Tatar O	American Journal of Ophthalmology	Expression of VEGF and PEDF in Choroidal Neovascular Membranes Following Verteporfin Photodynamic Therapy.	Non-*PEDF* gene
67	2006	Tong JP	American Journal of Ophthalmology	Aqueous humor levels of vascular endothelial growth factor and pigment epithelium-derived factor in polypoidal choroidal vasculopathy and choroidal neovascularization.	Non-*PEDF* gene
68	2006	Tsao YP	Life Sciences	Pigment epithelium-derived factor inhibits oxidative stress-induced cell death by activation of extracellular signal-regulated kinases in cultured retinal pigment epithelial cells.	Non-*PEDF* gene
69	2006	Yeoh J	Expert Opinion on Pharmacotherapy	A review of drug options in age-related macular degeneration therapy and potential new agents.	Non-*PEDF* gene
70	2007	Abe T	Progress in Retinal and Eye Research	Iris pigment epithelial cell transplantation for degenerative retinal diseases.	Non-*PEDF* gene
71	2007	Afzal A	Microvascular Research	Retinal and choroidal microangiopathies: Therapeutic opportunities.	Non-*PEDF* gene
72	2007	Campochiaro PA	Current Gene Therapy	Gene therapy for ocular neovascularization.	Gene therapy
73	2007	Damico FM	Arquivos brasileiros de oftalmologia	Angiogenesis and retinal diseases.	Review

74	2007	Emerson MV	BioDrugs	Emerging therapies for the treatment of neovascular age-related macular degeneration and diabetic macular edema.	Non-*PEDF* gene
75	2007	Gamulescu MA	Expert Opinion on Therapeutic Patents	A new era in the treatment of age-related macular degeneration: From factor X to antiangiogenesis.	Non-*PEDF* gene
76	2007	Hussain N	Indian Journal of Ophthalmology	The future implications and indications of anti-vascular endothelial growth factor therapy in ophthalmic practice.	Non-*PEDF* gene
77	2007	Lee SJ	Journal of Microbiology and Biotechnology	Human apolipoprotein E2 transgenic mice show lipid accumulation in retinal pigment epithelium and altered expression of VEGF and bFGF in the eyes.	Animal study
78	2007	Marra M	Expert Opinion on Drug Delivery	2nd Ophthalmic Drug Development and Delivery Summit San Diego, CA, USA, 19-20 September 2006.	Non-*PEDF* gene
79	2007	Rex TS	Ophthalmic Genetics	Rescue of sight by gene therapy - Closer than it may appear.	Non-*PEDF* gene
80	2007	Sherris D	Angiogenesis	Ocular drug development - Future Directions.	Non-*PEDF* gene
81	2007	Virgili G	Acta Ophthalmologica Scandinavica	New therapies for neovascular age-related macular degeneration: Critical appraisal of the current evidence.	Non-*PEDF* gene
82	2007	Wang SF	International Journal of Ophthalmology	Research progress of PEDF.	Review
83	2007	Yoshida T	Nippon Ganka Gakkai Zasshi - Acta Societatis Ophthalmologicae Japonicae	Molecular mechanism of choroidal neovascularization in age-related macular degeneration.	Non-*PEDF* gene
84	2007	Yu Y	Chinese Ophthalmic Research	Cloning of rat PEDF gene and construction of its adenovirus expression vector.	Animal study
85	2007	Zamiri P	Immune Response and the Eye	Immunosuppressive properties of the pigmented epithelial cells and the subretinal space.	Non-*PEDF* gene
86	2007	Zarbin M	Optometry and Vision Science	Current treatment of age-related macular degeneration.	Non-*PEDF* gene
87	2007	Zhang SX	Progress in Retinal and Eye Research	Ocular neovascularization: Implication of endogenous angiogenic inhibitors and potential therapy.	Non-*PEDF* gene
88	2008	Bhutto IA	Archives of Ophthalmology	Reduction of endogenous angiogenesis inhibitors in bruch's membrane of the submacular region in eyes with age-related macular degeneration.	Non-*PEDF* gene

89	2008	Chan WM	Retina (Philadelphia, Pa)	Changes in aqueous vascular endothelial growth factor and pigment epithelial-derived factor levels following intravitreal bevacizumab injections for choroidal neovascularization secondary to age-related macular degeneration or pathologic myopia.	Non-*PEDF* gene
90	2008	Chappelow AV	Drugs	Neovascular age-related macular degeneration: Potential therapies.	Non-*PEDF* gene
91	2008	Cryan LM	Proteomics - Clinical Applications	Proteomics as a research tool in clinical and experimental ophthalmology.	Non-*PEDF* gene
92	2008	Ehrlich R	Clinical Interventions in Aging	Age-related macular degeneration and the aging eye.	Non-*PEDF* gene
93	2008	Grisanti S	Progress in Retinal and Eye Research	The role of vascular endothelial growth factor and other endogenous interplayers in age-related macular degeneration.	Review
94	2008	Kaiser PK	Core Evidence	Ranibizumab: The evidence of its therapeutic value in neovascular age-related macular degeneration.	Non-*PEDF* gene
95	2008	Matsuno K	Folia Pharmacologica Japonica	Current status and future strategies for age-related macular degeneration.	Review
96	2008	McVey D	Molecular Therapy	Repeat administration of proteins to the eye with a single intraocular injection of an adenovirus vector.	Non-*PEDF* gene
97	2008	Penn JS	Progress in Retinal and Eye Research	Vascular endothelial growth factor in eye disease.	Non-*PEDF* gene
98	2008	Steinle JJ	Growth Factors	Beta-adrenergic receptor regulation of growth factor protein levels in human choroidal endothelial cells.	Non-*PEDF* gene
99	2009	Ablonczy Z	Journal of Biological Chemistry	Pigment epithelium-derived factor maintains retinal pigment epithelium function by inhibiting vascular endothelial growth factor-R2 signaling through -secretase.	Non-*PEDF* gene
100	2009	Ahn JK	American journal of ophthalmology	Changes in aqueous vascular endothelial growth factor and pigment epithelium-derived factor after ranibizumab alone or combined with verteporfin for exudative age-related macular degeneration.	Non-*PEDF* gene
101	2009	Dadgostar H	Expert Review of Ophthalmology	SiRNA therapeutics for agerelated macular degeneration: Promises and pitfalls.	Non-*PEDF* gene
102	2009	Dixon JA	Expert Opinion on Investigational Drugs	VEGF Trap-Eye for the treatment of neovascular age-related macular degeneration	Non-*PEDF* gene

103	2009	Hubschman JP	Clinical Ophthalmology	Age-related macular degeneration: Experimental and emerging treatments	Review
104	2009	Ikeda Y	Human Gene Therapy	Acute toxicity study of a simian immunodeficiency virus-based lentiviral vector for retinal gene transfer in nonhuman primates	Non-*PEDF* gene
105	2009	Joussen AM	Deutsches Arzteblatt	The treatment of wet age-related macular degeneration	Non-*PEDF* gene
106	2009	Lai TYY	Retina	Visual outcomes and growth factor changes of two dosages of intravitreal bevacizumab for neovascular age-related macular degeneration: A randomized, controlled trial	Non-*PEDF* gene
107	2009	Ni Z	Ophthalmologica	Emerging pharmacologic therapies for wet age-related macular degeneration	Non-*PEDF* gene
108	2009	Stahl A	Graefe's Archive for Clinical and Experimental Ophthalmology	Combinatory inhibition of VEGF and FGF2 is superior to solitary VEGF inhibition in an in vitro model of RPE-induced angiogenesis	Non-*PEDF* gene
109	2009	Thomson H	Nanomedicine	The promise of nanomedicine for ocular disease	Review
110	2009	Zhang P	Bioscience Hypotheses	Choroidal neovascularization in age-related macular degeneration depends on vascular endothelial growth factor, but vascular endothelial growth factor should not be the promising treatment target	Non-*PEDF* gene
111	2010	Anderson OA	Drug Discovery Today	Delivery of anti-angiogenic molecular therapies for retinal disease	Non-*PEDF* gene
112	2010	Balasubbu S	BMC Medical Genetics	Association analysis of nine candidate gene polymorphisms in Indian patients with type 2 diabetic retinopathy	Non-AMD or PCV
113	2010	Canavese M	Journal of Dermatological Science	Vascular endothelial growth factor (VEGF) in the pathogenesis of psoriasis-A possible target for novel therapies	Non-*PEDF* gene
114	2010	Colella P	Current Gene Therapy	AAV-Mediated Gene Supply for Treatment of Degenerative and Neovascular Retinal Diseases	Non-*PEDF* gene
115	2010	Hou HY	Chinese Journal of Ophthalmology	Progress on study of treatment of age-related macular degeneration by pigment epithelial-derived factor	Non-*PEDF* gene
116	2010	Imai D	Journal of Ocular Biology, Diseases, and Informatics	CFH, VEGF, and PEDF genotypes and the response to intravitreous injection of bevacizumab for the treatment of age-related macular degeneration	PEDF gene and treatment response

117	2010	Knepper JE	Molecular Biology of the Cell	Direct interaction of recombinant pigment epithelium derived factor PEDF with vascular endothelial growth factor receptors 1 and 2	Non-*PEDF* gene
118	2010	Laude A	Progress in Retinal and Eye Research	Polypoidal choroidal vasculopathy and neovascular age-related macular degeneration: Same or different disease	Review
119	2010	Murakami Y	Human Gene Therapy	Inhibition of choroidal neovascularization via brief subretinal exposure to a newly developed lentiviral vector pseudotyped with sendai viral envelope proteins	Non-*PEDF* gene
120	2010	Parmeggiani F	Mediators of Inflammation	Inflammatory mediators and angiogenic factors in choroidal neovascularization: Pathogenetic interactions and therapeutic implications	Non-*PEDF* gene
121	2010	Sonoda S	Aging	Attainment of polarity promotes growth factor secretion by retinal pigment epithelial cells: relevance to age-related macular degeneration	Non-*PEDF* gene
122	2010	Strunnikova NV	Hum Mol Genet	Transcriptome analysis and molecular signature of human retinal pigment epithelium	Non-*PEDF* gene
123	2010	Thumann G	Gene Therapy	High efficiency non-viral transfection of retinal and iris pigment epithelial cells with pigment epithelium-derived factor	Non-*PEDF* gene
124	2010	Tombran-Tink J	Current Molecular Medicine	PEDF in angiogenic eye diseases	Review
125	2011	Alghadyan AA	Saudi Journal of Ophthalmology	Diabetic retinopathy - An update	Non-AMD or PCV
126	2011	Bhise NS	Expert Opinion on Drug Delivery	Drug delivery strategies for therapeutic angiogenesis and antiangiogenesis.	Non-*PEDF* gene
127	2011	Campochiaro PA	Human Gene Therapy	Gene transfer for neovascular age-related macular degeneration	Non-*PEDF* gene
128	2011	De Dias JRO	British Journal of Ophthalmology	Cytokines in neovascular age-related macular degeneration: Fundamentals of targeted combination therapy	Non-*PEDF* gene
129	2011	Federici TJ	Pharmacological Research	The non-antibiotic properties of tetracyclines: Clinical potential in ophthalmic disease	Non-*PEDF* gene
130	2011	Kolomeyer AM	Investigative Ophthalmology and Visual Science	Characterization of conditioned media collected from aged versus young human eye cups	Non-*PEDF* gene

131	2011	Liu MM	Postgraduate Medical Journal	Republished review: Gene therapy for ocular diseases	Non-*PEDF* gene
132	2011	Liu MM	British Journal of Ophthalmology	Gene therapy for ocular diseases	Gene therapy
133	2011	Markovets AM	PloS one	Therapeutic action of the mitochondria-targeted antioxidant SkQ1 on retinopathy in OXYS rats linked with improvement of VEGF and PEDF gene expression	Non-*PEDF* gene
134	2011	Markovets AM	Aging	Alterations of retinal pigment epithelium cause AMD-like retinopathy in senescence-accelerated OXYS rats	Animal study
135	2011	Milward L	American Journal of Hematology	Iron and the biogenesis of melanin and melanosomes in the retina	Non-*PEDF* gene
136	2011	Nakata I	Ophthalmology	Genetic variants in pigment epithelium-derived factor influence response of polypoidal choroidal vasculopathy to photodynamic therapy	*PEDF* gene and treatment response
137	2011	Ng TK	Investigative Ophthalmology and Visual Science	Interactive expressions of HtrA1 and VEGF in human vitreous humors and fetal RPE cells	Non-*PEDF* gene
138	2011	Pons M	Investigative Ophthalmology and Visual Science	Nicotine increases the VEGF/PEDF ratio in retinal pigment Epithelium: A possible mechanism for CNV in passive smokers with AMD	Non-*PEDF* gene
139	2011	Pons M	PloS one	Cigarette smoke-related hydroquinone dysregulates MCP-1, VEGF and PEDF expression in retinal pigment epithelium in vitro and in vivo	Non-*PEDF* gene
140	2011	Rohrer B	Molecular Immunology	The alternative pathway is required, but not alone sufficient, for retinal pathology in mouse laser-induced choroidal neovascularization	Non-*PEDF* gene
141	2011	Schiffelers RM	BioDrugs	Neovascular age-related macular degeneration: Opportunities for development of first-in-class biopharmaceuticals	Non-*PEDF* gene
142	2011	Silva RA	Seminars in Ophthalmology	Radiation treatment for age-related macular degeneration	Non-*PEDF* gene
143	2011	Thumann G	Expert Review of Ophthalmology	Nonviral gene therapy for age-related macular degeneration	Gene therapy
144	2011	Truong A	Journal of Molecular Medicine	Emerging therapeutic approaches in the management of retinal angiogenesis and edema	Non-*PEDF* gene

145	2011	Tsuchihashi T	Ophthalmology	Complement factor H and high-temperature requirement A-1 genotypes and treatment response of age-related macular degeneration	Non-*PEDF* gene
146	2011	Wen F	[Chinese]. Zhonghua Shiyan Yanke Zazhi/Chinese Journal of Experimental Ophthalmology	Current researches and existing problems of molecular biology in neovascular age-related macular degeneration and polypoidal choroidal vasculopathy	Review
147	2011	Wornle M	Investigative ophthalmology & visual science	Inhibition of TLR3-mediated proinflammatory effects by Alkylphosphocholines in human retinal pigment epithelial cells	Non-*PEDF* gene
148	2011	Yin L	Current Eye Research	OX-LDL up-regulates the vascular endothelial growth factor-to-pigment epithelium-derived factor ratio in human retinal pigment epithelial cells	Non-*PEDF* gene
149	2011	Zhu D	Investigative Ophthalmology and Visual Science	Polarized secretion of PEDF from human embryonic stem cell-derived RPE promotes retinal progenitor cell survival	Non-*PEDF* gene
150	2012	Anna M	Klinika Oczna	PEDF and VEGF plasma level alterations in patients with dry form of age-related macular degeneration - A possible link to the development of the disease	Non-*PEDF* gene
151	2012	Bai YJ	Journal of Pharmacology and Experimental Therapeutics	Polyethylene glycol-modified pigment epithelial-derived factor: New prospects for treatment of retinal neovascularization	Non-*PEDF* gene
152	2012	Bandyopadhyay M	Investigative ophthalmology & visual science	Matrix metalloproteinase activity creates pro-angiogenic environment in primary human retinal pigment epithelial cells exposed to complement	Non-*PEDF* gene
153	2012	Cai X	Retinal Degenerative Diseases	Neovascularization: Ocular diseases, animal models and therapies	Review
154	2012	Cunnusamy K	Pharmaceutical Patent Analyst	Next-generation therapeutic solutions for age-related macular degeneration	Non-*PEDF* gene
155	2012	Huber M	Ophthalmologica	Vitreous levels of proteins implicated in angiogenesis are modulated in patients with retinal or choroidal neovascularization	Non-*PEDF* gene

156	2012	Johnen S	Investigative Ophthalmology and Visual Science	Sleeping Beauty transposon-mediated transfection of retinal and iris pigment epithelial cells	Non-*PEDF* gene
157	2012	Kim LA	Current Angiogenesis	Neovascular age-related macular degeneration: Past, present, and future	Review
158	2012	Machalinska A	Klinika Oczna	PEDF and VEGF plasma level alterations in patients with dry form of age-related degeneration--a possible link to the development of the disease	Non-*PEDF* gene
159	2012	Nagineni CN	Journal of Cellular Physiology	Regulation of VEGF expression in human retinal cells by cytokines: Implications for the role of inflammation in age-related macular degeneration	Non-*PEDF* gene
160	2012	Nowak-Sliwinska P	Current Angiogenesis	Anti-angiogenic treatment for exudative age-related macular degeneration: New strategies are underway	Non-*PEDF* gene
161	2012	Sekiyama E	FASEB Journal	Heat treatment of retinal pigment epithelium induces production of elastic lamina components and antiangiogenic activity	Non-*PEDF* gene
162	2012	Smith GW	Retinal Degenerative Diseases	The importance of hypoxia-regulated, RPE-targeted gene therapy for choroidal neovascularization	Non-*PEDF* gene
163	2012	Thumann G	Current Genomics	Prospectives for gene therapy of retinal degenerations	Gene therapy
164	2013	Al-Shabrawey M	Expert Review of Ophthalmology	Targeting neovascularization in ischemic retinopathy: Recent advances	Non-*PEDF* gene
165	2013	Bianchi E	International Journal of Molecular Medicine	Retinal pigment epithelium, age-related macular degeneration and neurotrophic keratouveitis	Non-*PEDF* gene
166	2013	Buchholz DE	Stem Cells Translational Medicine	Rapid and efficient directed differentiation of human pluripotent stem cells into retinal pigmented epithelium	Non-*PEDF* gene
167	2013	Craword SE	Expert Opinion on Drug Discovery	The many facets of PEDF in drug discovery and disease: A diamond in the rough or split personality disorder	Non-*PEDF* gene
168	2013	Kolomeyer AM	Graefe's Archive for Clinical and Experimental Ophthalmology	Characterization of the effects of retinal pigment epithelium-conditioned media on porcine and aged human retina	Non-*PEDF* gene
169	2013	Kuo JZ	Expert Review of Ophthalmology	Genetic risk, ethnic variations and pharmacogenetic biomarkers in AMD and polypoidal choroidal vasculopathy	Review
170	2013	Sivaprasad S	British Medical Bulletin	What is new in the management of wet age-related macular degeneration	Non-*PEDF* gene

171	2013	Suen WLL	Journal of Controlled Release	Specific uptake of folate-decorated triamcinolone-encapsulating nanoparticles by retinal pigment epithelium cells enhances and prolongs antiangiogenic activity	Non-*PEDF* gene
172	2014	Askou AL	Acta Ophthalmologica	Development of gene therapy for treatment of age-related macular degeneration	Gene therapy
173	2014	Croze RH	Stem Cells Translational Medicine	ROCK inhibition extends passage of pluripotent stem cell-derived retinal pigmented epithelium	Non-*PEDF* gene
174	2014	Duggan C	Cancer Epidemiology Biomarkers and Prevention	Effect of a 12-month exercise intervention on serum biomarkers of angiogenesis in postmenopausal women: A randomized controlled trial	Non-*PEDF* gene
175	2014	Kolomeyer AM	Survey of Ophthalmology	Trophic factors in the pathogenesis and therapy for retinal degenerative diseases	Non-*PEDF* gene
176	2014	McHugh KJ	Investigative Ophthalmology and Visual Science	Porous poly(-caprolactone) scaffolds for retinal pigment epithelium transplantation	Non-*PEDF* gene
177	2014	Pastor M	Molecular Therapy	The merging of the antibiotic-free pFAR4 miniplasmids with the sleeping beauty transposon system mediates higher transgene delivery in human cells	Non-*PEDF* gene
178	2014	Payne AJ	International Journal of Molecular Sciences	Antioxidant drug therapy approaches for neuroprotection in chronic diseases of the retina	Non-*PEDF* gene
179	2014	Shen W	Glia	Effect of glucocorticoids on neuronal and vascular pathology in a transgenic model of selective Muller cell ablation	Non-*PEDF* gene
180	2014	Smith AG	Expert Opinion on Emerging Drugs	Emerging treatments for wet age-related macular degeneration	Non-*PEDF* gene
GWAS excluded study					
1	2010	Neale BM	Proceedings of the National Academy of Sciences	Genome-wide association study of advanced age-related macular degeneration identifies a role of the hepatic lipase gene (LIPC).	Not provide genotype or allele data in controls

Table S6. Risk of bias assessment for AMD and PCV genetic association studies

Criteria	Assessment
Information bias	
Ascertainment of AMD and PCV	
Clearly diagnostic criteria of AMD and PCV (FFA and ICGA confirmed)	Yes
Not clearly description (only FFA or ICGA confirmed)	No
Not mention	Unclear
Ascertainment of controls	
Non-AMD and non-PCV controls with ocular examination	Yes
Non-AMD and non-PCV controls without ocular examination	No
Not description	Unclear
Confounding bias	
Population stratification	
No ethnic difference between cases and controls	Yes
Unrelated Controls without AMD and PCV	Yes
Some controls from the same family	No
Not description	Unclear
Other confounding bias	
Controlled for confounding factors (e.g. gender, age, smoking)	Yes
Not controlled for confounding factors	No
Not description	Unclear
Hardy-Weinberg equilibrium in controls	
Hardy-Weinberg equilibrium	Yes
Hardy-Weinberg disequilibrium	No
Not mention	Unclear

AMD: age-related macular degeneration; FFA: fundus fluorescein angiography; ICGA: indocyanine green angiography; PCV: polypoidal choroidal vasculopathy.

Study or Subgroup	PCV Events	PCV Total	Control Events	Control Total	Weight	Odds Ratio M-H, Fixed, 95% CI
Bessho H 2009 (Japanese)	138	280	175	378	44.2%	1.13 [0.83, 1.54]
Wu K 2012 (Chinese)	182	354	199	364	55.8%	0.88 [0.65, 1.18]
**Total (95% CI)**		**634**		**742**	**100.0%**	**0.99 [0.80, 1.22]**
Total events	320		374			

Heterogeneity: Chi² = 1.33, df = 1 (P = 0.25); I² = 25%
Test for overall effect: Z = 0.11 (P = 0.91)

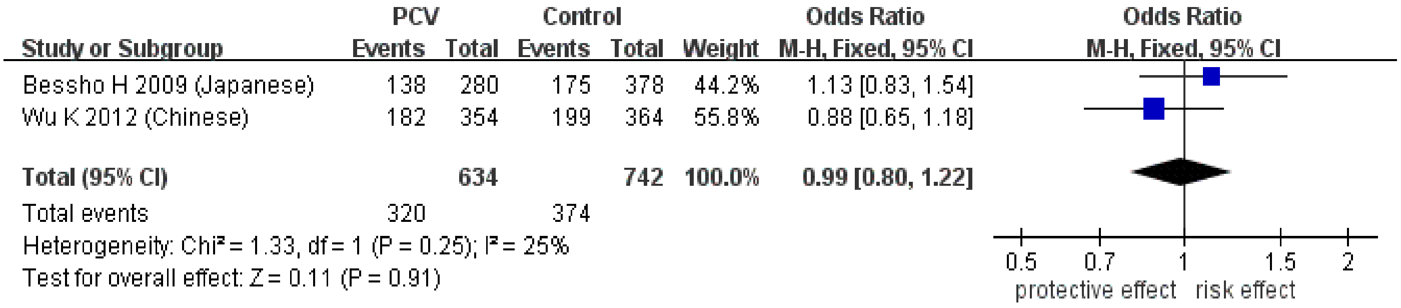

Figure S1. Forest plot of rs1136287(T) in PCV in allelic model. Squares indicate study-specific odds ratios (ORs). The size of the box is proportional to the weight of the study. Horizontal lines indicate 95% confidence intervals (CI). A diamond indicates the summary OR with its corresponding 95% CI. PCV: polypoidal choroidal vasculopathy.

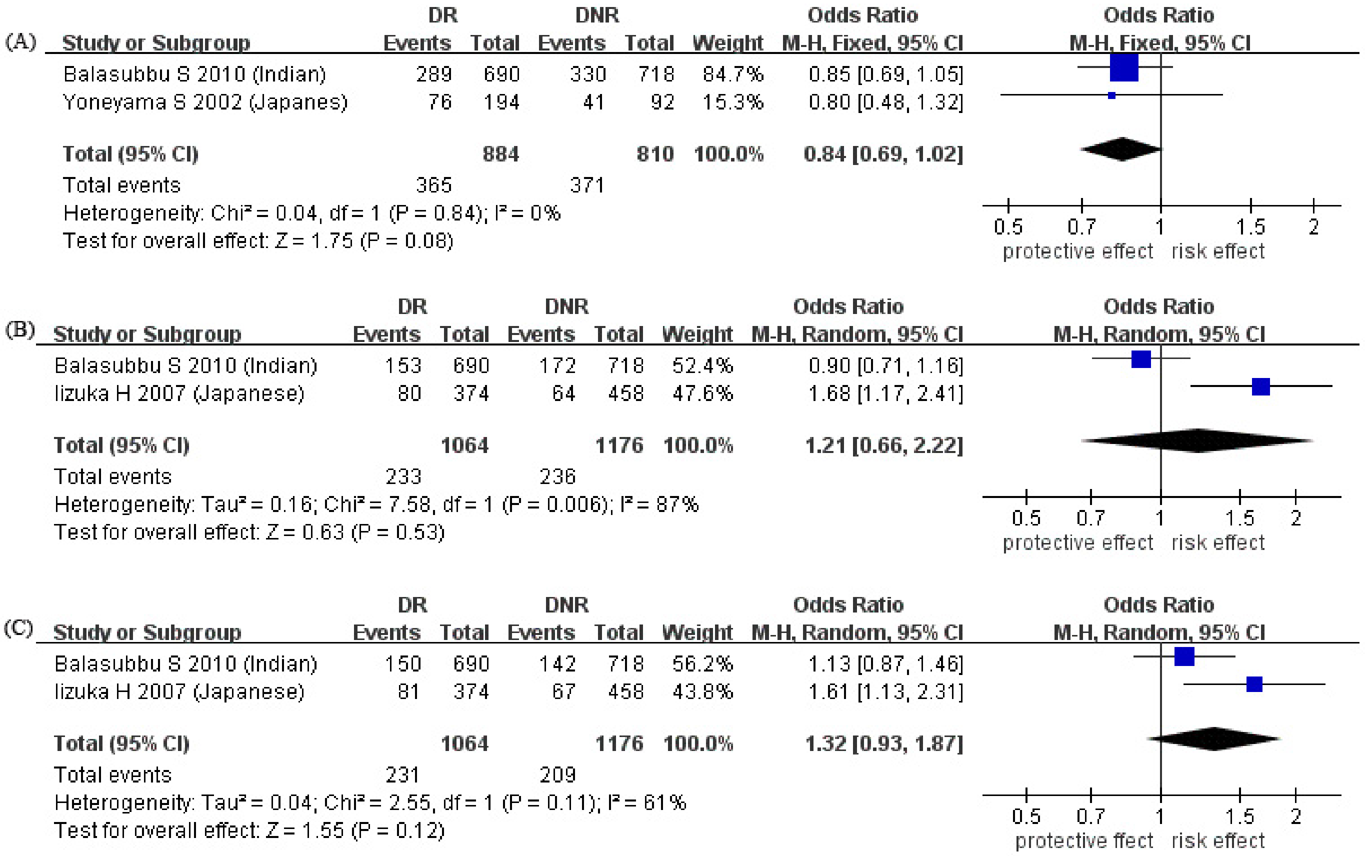

Figure S2. Forest plots in DR in allelic model. (A) Forest plot of rs1136287(T); (B) Forest plot of rs12150053(C); (C) Forest plot of rs12948385(A). Squares indicate the study-specific odds ratios (ORs). The size of the box is proportional to the weight of the study. Horizontal lines indicate 95% confidence intervals (CI). A diamond indicates the summary OR with its corresponding 95% CI. DR: diabetic retinopathy.
